# How the Global South is reshaping scholarly communication

**DOI:** 10.7554/eLife.108426

**Published:** 2025-09-01

**Authors:** Humberto Debat, Izuchukwu Azuka Okafor, Mercury Shitindo, Olavo B Amaral, Nurul Izzati, Chandana Basu Mallick, Awika O Henry, Roseline Dzekem Dine, Carolina Santacruz-Perez

**Affiliations:** 1 National Institute of Agricultural Technology Córdoba Argentina; 2 Department of Molecular Biology, University Medical Center Göttingen Göttingen Germany; 3 Nnamdi Azikiwe University Awka Nigeria; 4 UMass Chan Medical School Worcester United States; 5 Africa Bioethics Network Nairobi Kenya; 6 University of Zaragoza Zaragoza Spain; 7 Federal University of Rio de Janeiro Rio de Janeiro Brazil; 8 Sumbawa University of Technology Sumbawa Indonesia; 9 Banaras Hindu University Varanasi India; 10 University of the Virgin Islands St Croix United States; 11 Rinda Ubuzima Kigali Rwanda; 12 Review and Curate Network Kigali Rwanda; 13 International Science Council Paris France

**Keywords:** global south, open access, open science, research assessment, scientific publishing, point of view, None

## Abstract

The Global South has emerged as a beacon of innovation in scholarly publishing, championing equitable and community-driven models of knowledge dissemination. Here we explore various initiatives originating from Latin America, Africa, and Asia, highlighting platforms that are free for both readers and authors. These initiatives also promote multilingualism, public funding, and non-profit, scholarly-led governance structures. We argue that the Global South’s approach offers a blueprint for a fairer, more inclusive, and more just academic publishing ecosystem.

In the world of scholarly publishing, where commercial publishers, high-impact journals, article processing charges, and the dominance of the English language often dictate who gets to participate in scientific dialogue (Buranyi, 2017), a quiet revolution has been unfolding across the Global South. While prevailing narratives paint open access mostly as a Northern innovation, the reality is that regions like Latin America, Africa, and Asia have long been building their own vibrant, community-centered scholarly ecosystems, offering a vision of publishing that is equitable, multilingual, and open (Debat and Babini, 2020).

The transition from print to digital formats in the late 20th century brought both opportunities and challenges. For many in the Global North, it meant new revenue streams for commercial publishers, while in the Global South, it led to the creation of a new infrastructure for the dissemination of knowledge in a way that was equitable and inclusive, without paywalls or profit motivations. Fueled by necessity, collaboration, and public funding, a growing number of initiatives in the Global South have helped to redefine scholarly communication ensuring equity and public good (Babini, 2019). Previously we have highlighted the leadership of Latin America and other regions of the Global South in creating public, non-commercial models of open access models (Debat et al., 2025). Here we make a number of proposals for reclaiming the scholarly commons at the global level, inspired by principles of solidarity, equity, and multilingualism.

## The rise of diamond open access

One of the most remarkable contributions of the Global South to scholarly communication has been its pioneering role in diamond open access, a model in which publications are free to read and free to publish in. Long before diamond open access started to gain traction in Europe or North America, Latin American initiatives like SciELO, Redalyc, and CLASCO built public infrastructures to ensure that research outputs remain publicly and widely accessible and regionally relevant (Cetto et al., 2015).

SciELO, launched in Brazil in 1997, is perhaps the most emblematic of these efforts (SciELO, 2015). Initially funded by São Paulo’s state science agency, later embraced by the Brazilian government, and now expanded across multiple countries, SciELO offers free access to hundreds of journals while also enforcing quality standards, indexing protocols, and open science practices. Its decentralized structure, where each participating country manages its collection, preserves autonomy while fostering regional interoperability.

Similarly, Redalyc in Mexico and the continent-wide initiative AmeliCA emerged with a clear mission: to offer an alternative to the pay-to-publish model and strengthen the role of non-commercial, scholarly-led journals (Torres Samuel and Matute, 2022). These platforms work not just as repositories but as strategic interventions against a growing commodification of academic publishing. As a result, thousands of journals across the Global South now operate without article processing charges (APCs), ensuring participation regardless of institutional wealth or researcher prestige (Khanna et al., 2022). Notable examples include *Memórias do Instituto Oswaldo Cruz*, published by Fiocruz in Brazil and indexed in SciELO, which is internationally recognized in parasitology and tropical medicine. Likewise, *Revista Austral de Ciencias Sociales*, based in Chile and indexed in Redalyc, has become a leading venue for social science research in the Southern Cone and broader Latin American region.

These platforms also challenge the English-language hegemony by promoting multilingual publishing and supporting indigenous and regional languages. Where most Northern platforms default to English, SciELO and AmeliCA also provide peer review and editorial support in Spanish, Portuguese, and increasingly, native languages, with several journals including multilingual articles, making room for diverse epistemologies to thrive.

## Beyond Latin America: A global mosaic of innovation

Africa’s scholarly communications landscape has likewise evolved through necessity and creativity. African Journals Online (AJOL), founded in 1998, remains the continent’s largest open-access platform. Today, it hosts over 500 journals, giving global visibility to African research across disciplines.

At a systems level, the African Open Science Platform (AOSP) and regional networks like the West and Central African Research and Education Network (WACREN), the Arab States Research and Education Network (ASREN), and UbuntuNet Alliance are building infrastructure that connects universities and researchers across borders. They support repository development, promote FAIR (short for findable, accessible, interoperable and reusable) data principles, and advance health-focused initiatives like the Coalition for Open Access Publishing of Public Health in Africa (COPPHA).

Asia tells yet another story. From Shodhganga (an open-access thesis repository) in India to GARUDA in Indonesia and ThaiLIS in Thailand, national governments and education ministries are driving the creation of integrated platforms that emphasize local relevance, inclusion, open access, and capacity building. China has advanced open access through platforms like ChinaXiv (a preprint server for natural sciences), NCPSSD (which contains over 26 million documents, related to philosophy and social sciences), PubScholar (which provides free access to approximately 170 million academic resources), and OpenSign (an open platform aggregating over 10 million global open access papers to facilitate academic discovery and sharing).

These efforts converge on a shared goal: to facilitate knowledge dissemination within the Global South and resist epistemic dependence on Northern infrastructures (Table 1).

**Table 1.** Summary of the scholarly communication platforms and repositories mentioned in this article. For each platform/repository the table lists the following information: URL; year of launch; number of journals, articles and preprints; disciplinary coverage; funding sources; and region. Data were compiled from websites and/or publicly available reports during August 2025.

*Name*	*URL*	*Launch*	*Number of journals, articles and preprints*	*Disciplines*	*Funding source*	*Region*
SciELO SciELO Preprints	https://scielo.org/, https://preprints.scielo.org/	1997/98 2020	1,654 journals;~693k OA articles.~4k preprints.	Multi-disciplinary	Funded by public research funding agencies in Brazil	Latin America
Redalyc	https://www.redalyc.org/	2002	1,727 journals; over 782k full-text OA articles.	Social & Natural Sciences	Academic-led, nonprofit; hosted at UNAM, Mexico; supported via university grants.	Latin America
CLACSO	https://www.clacso.org/	1998	1,060 journals;~479k articles	Multi-disciplinary	Publicly funded mostly by UNESCO.	Latin America
AfricArXiv	http://info.africarxiv.org/	2018	480 preprints; 543 publications in ScienceOpen collection.	Multi-disciplinary	Community-led, supported by Center for Open Science.	Africa
ChinaXiv	https://chinaxiv.org/	2016	~43k preprint articles.	Multi-disciplinary	Publicly funded Chinese-language preprint platform	Asia
NCPSSD	http://www.ncpssd.cn/	2016	2,467 journals and more than 26 million articles.	Philosophy & Social Sciences	Publicly funded Chinese national repository	Asia
PubScholar	https://pubscholar.cn/	2023	~95 million journal articles and ~3.6 million indexed preprints.	Multi-disciplinary	Publicly funded repository of Chinese Academy of Sciences outputs	Asia
OpenSign	https://opensign.lib.tsinghua.edu.cn/	2024	37,810 journals and ~10 million OA indexed articles.	Multi-disciplinary	Publicly funded aggregated OA search platform by Tsinghua University	Asia
Shodhganga	https://shodhganga.inflibnet.ac.in/	2009	~620k theses from ~840 universities.	Theses & Dissertations (India)	Publicly funded national open-access thesis repository	Asia
GARUDA	https://garuda.kemdikbud.go.id/	2010	More than 1 million articles, and journals covering almost 40 fields of science.	Multi-disciplinary (Indonesia)	Government-backed platform	Asia
ThaiLIS	https://tdc.thailis.or.th/tdc/	2004	~150k theses and ~17k articles.	Multi-disciplinary (Thailand)	Publicly funded National library initiative	Asia
African Journals OnLine (AJOL)	http://www.ajol.info/	1998	~263k journal articles from 893 OA journals.	Multi-disciplinary	Non-Profit Organisation funded by donors.	Africa
African Open Science Platform	https://aosp.org.za/	2017	Data not available.	Multi-disciplinary	Publicly funded	Africa

## Changing the North from the South

While these regional efforts were born out of necessity, their ripple effects are increasingly global. The European funders behind cOAlition S, an initiative to enforce full and immediate open access, have acknowledged SciELO and Redalyc as early models for diamond open access (Stern et al., 2023). Their proposal to shift from commercial APC-driven models to community-funded approaches as a more responsible publishing strategy owes much to these Southern precedents.

Preprint platforms such as Chinaxiv in Asia, SciELO Preprints in Latin America and AfricArXiv in Africa have also become important tools for the early dissemination of research in the Global South, in a way that is inclusive and supports multilingual submissions and regional visibility. The growth of these platforms reflects a broader adoption of preprint platforms, notably bioRxiv and medRxiv. The advantages of preprints – they are free for authors and readers, they are fast, and they allow authors to receive feedback on their work – are especially relevant for researchers in regions where access to traditional publishing is limited.

Moreover, metrics themselves are beginning to change. Southern initiatives such as the Latin American Forum for Research Assessment (FOLEC) are on a path to decentralize and foster measures of research based not on prestige or impact factor but on policy influence, societal benefit, and use by NGOs or community actors. FOLEC’s Declaration of Principles (2022), endorsed by over 270 institutions, explicitly calls for evaluation criteria that reward publications in diamond open-access journals and multilingual outputs, and encourages the integration of regional and local databases alongside international indexes. FOLEC also advocates for the development and use of ‘socio-territorial’ metrics which assess scientific contributions based on community engagement, regional policy impact, and cultural relevance, rather than traditional journal‐based citation counts. These emergent models offer an alternative to traditional citation-based metrics, aligning with global reform movements like DORA (Declaration on Research Assessment), CoARA (Coalition to Advance Research Assessment), and the Hong Kong Principles (Moher et al., 2020).

## Confronting structural challenges

Despite their regional successes, open-access ecosystems in the Global South face substantial hurdles. Underfunded infrastructure and limited internet capabilities continue to be a problem. Preservation is another concern. Many institutions lack the digital infrastructure or financial capacity to maintain long-term repositories, raising the specter of data loss. Initiatives are often fragmented, with duplicative or siloed efforts weakening regional and national coherence.

Research assessment and incentives continue to be problematic. An over-reliance on foreign indexing systems that privilege commercial publishers (such as Scopus or Web of Science; Kulczycki et al., 2025), and on international university rankings, continue to marginalize Southern scholarship (Clair, 2021). And academic reward systems, often imported from the Global North, still favor publishing in high-impact, English-language journals, although the extent to which this happens varies from country to country (Lim et al., 2025). These practices can discourage researchers from submitting to regional or national platforms, even when those platforms offer greater accessibility and a more relevant readership.

The persistence of these problems reflects a systemic feedback loop involving multiple actors: university administrators who rely on global rankings and impact metrics for evaluation; funders who tie grants to such indicators; and researchers who respond to these incentives to advance their careers. Addressing these issues requires coordinated reform across institutions, funders, and academic cultures to recognize diverse and locally relevant scholarly contributions.

Reforming these incentives is essential, and any assessment of research should extend beyond metrics and impact factors to include community engagement, policy influences, and contributions to regional challenges, from tropical diseases to food security. Without such a shift, open-access platforms will remain undervalued and underused (Cetto et al., 2015).

## Going forward: Practical recommendations

To build equitable and sustainable scholarly communication systems, the Global South must adopt practical strategies rooted in local priorities.

First, long-term public funding is essential. Brazil’s nationalization of SciELO illustrates how state investment can secure open access as strategic infrastructure (CAPES, 2024a).

Second, regional collaboration should be strengthened. Initiatives like SciELO, Redalyc, and La Referencia show the value of transnational, South–South networks grounded in shared goals. In addition to providing infrastructure for open-access publishing and indexing, the platforms also foster interoperability, multilingualism, and policy alignment across countries, demonstrating how regional cooperation can amplify visibility and autonomy in scholarly communication.

Third, North–South partnerships must respect local models. The backlash against the original Plan S in Latin America highlights the risks of imposing external frameworks. Recent shifts by COAlition S and the support of funders like Wellcome Trust and the Gates Foundation, suggest growing space for collaborative, equity-focused approaches.

Fourth, research assessment in the Global South must be reformed because the persistence of metrics like the Journal Impact Factor undermines many of the initiatives described in this article (Lim et al., 2025). There have already been moves in this direction. In Brazil, for example, the agency responsible for the evaluation of higher education institutions (CAPES) is replacing a system for assessing research outputs that relied on journal name (Qualis Periódicos) with a new system that relies on a mixture of quantitative and qualitative metrics to assess individual articles (CAPES, 2024b). Similar initiatives are needed across the Global South.

Finally, embracing open-source tools and emerging technologies, like Open Journal Systems and AI-enhanced translation, can reduce barriers to entry and boost visibility. Investing in technical capacity and innovation will be key to shaping an inclusive, resilient future for scholarly communication in the Global South.

## Reclaiming the scholarly commons

The future of scholarly publishing does not lie in reproducing the prestige-driven models of the past, but in building systems that reflect the world’s full intellectual diversity. That future is already being imagined in the Global South. Sustainable diamond open access models, publicly funded and governed by academic communities, offer a proven pathway to knowledge equity.

Scholarly communication is not just a technical enterprise, it also has political and ethical dimensions because it determines who gets to speak, what counts as knowledge, and whose challenges are prioritized. The Global South has shown that different systems are not only possible but are already thriving, built on solidarity, multilingualism, and public good principles (Babini, 2019). The time has come to recognize these initiatives not as exceptions or alternatives, but as models for the world. The next frontier of scholarly publishing should not be led by impact factors or corporate mergers. It should be shaped by those who see knowledge not as a commodity, but as a commons (UNESCO, 2021).

## Data Availability

There are no data associated with this article.
